# TagTrainer: supporting exercise variability and tailoring in technology supported upper limb training

**DOI:** 10.1186/1743-0003-11-140

**Published:** 2014-09-24

**Authors:** Daniel Tetteroo, Annick AA Timmermans, Henk AM Seelen, Panos Markopoulos

**Affiliations:** Department of Industrial Design, Eindhoven University of Technology, Eindhoven, Netherlands; Adelante Centre of Expertise in Rehabilitation and Audiology, Zandbergsweg 111, Hoensbroek, Netherlands; Department of Rehabilitation Medicine, Research School Caphri, Maastricht University, Universiteitssingel 40, Maastricht, 6229 Netherlands; Biomedical Research Institute, Faculty of Medicine and Life Sciences, Hasselt University, Martelarenlaan 42, Belgium, 3500 USA

## Abstract

**Background:**

Rehabilitation technology for upper limb training can potentially increase the amount, duration, and quality of therapy offered to patients by targeting the needs of individual patients. Empirical evaluations of such technologies focus on clinical effectiveness; however, little is known regarding the implications of their implementation in daily practice. Tailoring training content to patients requires active participation by therapists, and requires an extension of their role to include authoring and modifying exercises. It is not yet known whether this is feasible, and the socio-technical requirements that will make it successful in practice have not yet been explored. The current study investigates the extent to which therapists can take the role of authoring patient-specific training content and whether effort savings can be achieved by sharing the created content.

**Method:**

We present TagTrainer: an interactive tabletop system for rehabilitation that can be operated by manipulating every day physical objects in order to carry out exercises that simulate daily living tasks. TagTrainer supports therapists in creating their own exercises that fit individual patient needs, in adjusting existing exercises, and in putting together personalized exercise programs for and with patients. Four therapists in stroke- and paraplegia-rehabilitation have used TagTrainer for three weeks. Semi-structured interviews were conducted with the therapists, questionnaires were administered to them, and observation notes and usage logs were collected.

**Results:**

A total of 20 exercises were created from scratch, while another three exercises were created as variations of the existing ones. Importantly, all these exercises were created to address specific needs that patients expressed. The patients found the exercises motivating and these exercises were integrated into their regular training.

**Conclusions:**

TagTrainer can support arm-hand rehabilitation training by increasing therapy variability and tailoring. Therapists consider TagTrainer most suited for group sessions where they supervise many patients at once. Therapists are motivated and are able to, with minimal training, create and tailor exercises for patients fitting individual needs and capabilities. Future research will examine the socio-technical conditions that will encourage therapists to contribute and share training content, and provide the peer support needed for the adoption of a new technology.

**Electronic supplementary material:**

The online version of this article (doi:10.1186/1743-0003-11-140) contains supplementary material, which is available to authorized users.

## Background

Upper limb dysfunction has a major impact on many patients suffering from a neurological disease. For example, more than 40% of stroke survivors suffer from chronic upper extremity problems, which limits functional performance and engagement in community life [1, 2]. In addition, in tetraplegic patients, upper extremity impairment has a high impact on the patient’s functioning and is raised to the first priority for rehabilitation [3]. Rehabilitation can improve arm-hand performance after stroke and spinal cord injuries, and increasing exercise therapy intensity can improve treatment outcomes [4, 5]. However, offering increased training intensity to patients is hampered by the growing demand for resources on the health system, associated with current demographic trends and the need to address the growing incidences of spinal cord injuries [6] and strokes [7].

Technology-supported rehabilitation can potentially enable independent training with minimal therapist involvement. A literature survey of research on rehabilitation technology for arm-hand training [8] shows that the majority of this work has focused on impairment-based training and is not fully aligned with state-of-the-art trends in neurorehabilitation, which requires offering patient-tailored [9, 10] and task-oriented training [5, 11], [12]. Another important requirement is variation in training, which has been shown to contribute towards enhanced motor learning by increasing engagement and attention during learning [13], by allowing for random practice [11], and by offering a broader range of movement experiences, used in performing new skills [14]. Lack of exercise variability leads to a cessation of progress in patients because training offers no new challenges to them [15, 16].

Exercise variability and training content tailored to patients’ needs is essential in technology-supported training, in order to sustain compliance of patients to the therapy for longer periods. For example, a clinical trial of a sensor based technology that supports arm-hand training with real life objects for stroke patients, showed that very satisfactory treatment effects could be obtained, even in the chronic phase after the stroke [12]. Nevertheless, patients found that after eight weeks, the challenge had decreased. The patients indicated that, to keep training with the system, a higher number and a wider variety of exercises would be necessary.

Achieving exercise variability in technology-supported training is not trivial. Even in regular therapy, exercise variability is bounded by the expertise of therapists and other contextual constraints. Even more so, the range of exercises that can be created by a technology provider is bounded and current technologies offer quite a limited number of exercises/games to support training. A potential solution is for therapists themselves to be empowered to create exercises in technology supported training systems. This has the advantage that a therapist can create exercise content that fits the patient’s individual goals and ambitions, in line with the concepts of client-centred therapy, i.e. training where a patient can practise exercises that support his/her own training goals. Client-centred training increases patient motivation, patient self-efficacy, a patient’s training adherence, and consequently the effectiveness of the training program [9, 10]. Furthermore, a larger number of exercises can be made available depending on the creativity of the individual therapists [17] and the time available to them, while sharing and exchanging exercises among therapists can help patients benefit from diverse expertise and backgrounds.

Previous research has shown that technology acceptance [18] and self-efficacy [19] play an important role when it comes to implementing technology in the field of (neuro-) rehabilitation [20]. However, little is known about what factors can play a role in enabling therapists to become *creators* of technology supported training content.

The current paper presents an implementation study of TagTrainer, a new *end-user extensible* rehabilitation technology for arm-hand training, at a rehabilitation clinic. An action research approach was adopted in the current study. Action research combines both action and research within the same process and aims at generating knowledge by improving practice, and improving practice by the application of knowledge [21].

This study aims to identify whether and how therapists can successfully take the role of authoring training content, tailoring exercises to individual patients, and whether effort savings can be achieved by sharing the training content they create.

## Methods

### TagTrainer

**Figure 1:**
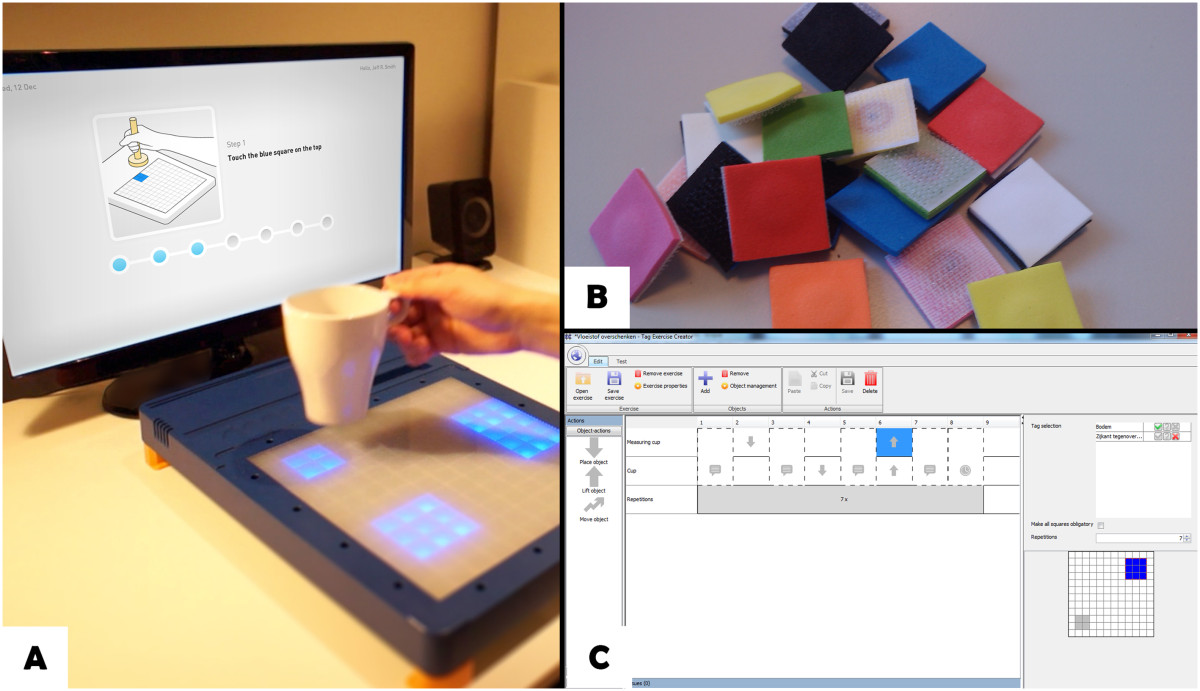
**Components of the TagTrainer system.** Patients perform training exercises on the TagTile board, while being guided by the TagTrainer Patient Interface **(A)**. Tagging physical objects with 2 x 2 cm sized RFID tags enables their use in TagTrainer exercises **(B)**. Exercises can be modified or created with the TagTrainer Exercise Creator **(C)**.

**Figure 2:**
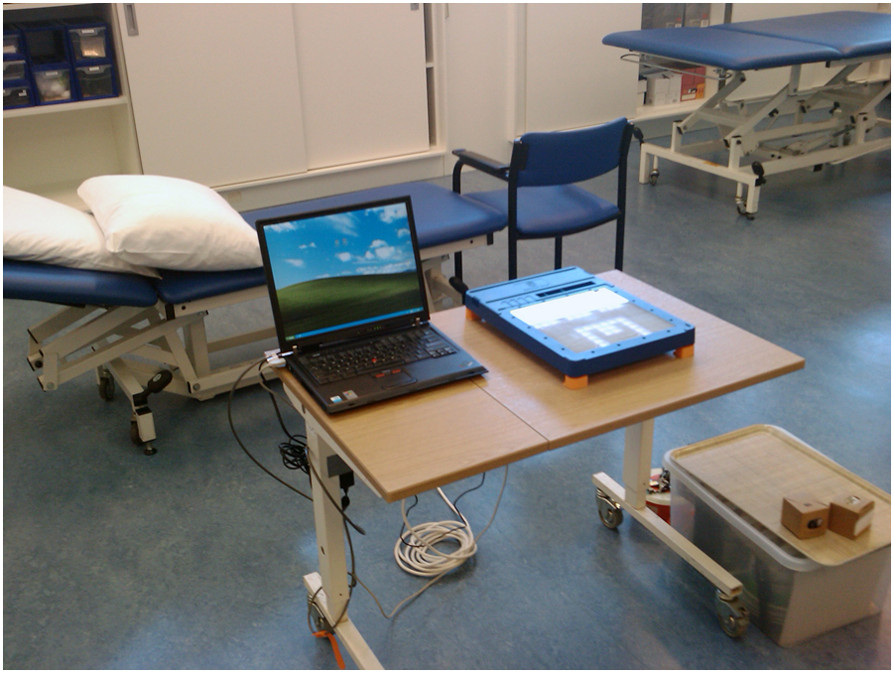
**A fully set-up TagTrainer system, ready for therapy.**

The TagTrainer system (see Figure 1) consists of an interactive board (TagTile, developed by Serious Toys BV, The Netherlands) and a laptop running accompanying software developed by the first author (DT). The TagTile board [22] is a programmable, interactive table top device that is able to detect and identify physical objects equipped with Radio Frequency Identification (RFID) tags, locate them on a grid (12 × 12 cells of 4 cm^2^ each), and provide both visual and auditory output. RGB LEDs on each cell of the grid allow the cells to be lit up, thus providing potential visual stimuli and feedback for exercises. Objects with one or more RFID tags (see Figure 1-B) can be used to interact with the board. During the study, one TagTrainer system was available for stroke rehabilitation purposes and one for spinal cord injury (SCI) rehabilitation. For both groups, the systems were mounted on a mobile, height-adjustable table (see Figure 2).

TagTrainer exercises typically include spoken instructions played back for the patient, and provide targets for different kinds of movements as lit areas on the board. When a tag is detected on the target, indicating that the corresponding object has been moved correctly, TagTrainer gives confirmatory feedback and sets new targets. An exercise is built up by choosing and tagging physical objects appropriately and planning a series of actions on the TagTile board, in order to elicit appropriate movements from the patient.

### Tag exercise creator

To address the challenge of creating exercises efficiently and with low costs, the Tag Exercise Creator (TEC) was developed: a software application written in Java that enables the creation of exercises without the need for extensive programming, using a simple graphical user interface (see Figure 1-C). TEC runs on a PC connected to the TagTile board. The TEC was developed in a user centred manner involving therapists throughout its conception and design, as well as an extensive user test with final year students in physiotherapy [23]. With the results from that test, as well as guidelines derived from previous research [24], the TEC was further developed to a level deemed sufficient for its initial implementation in daily therapy.

**Figure 3:**
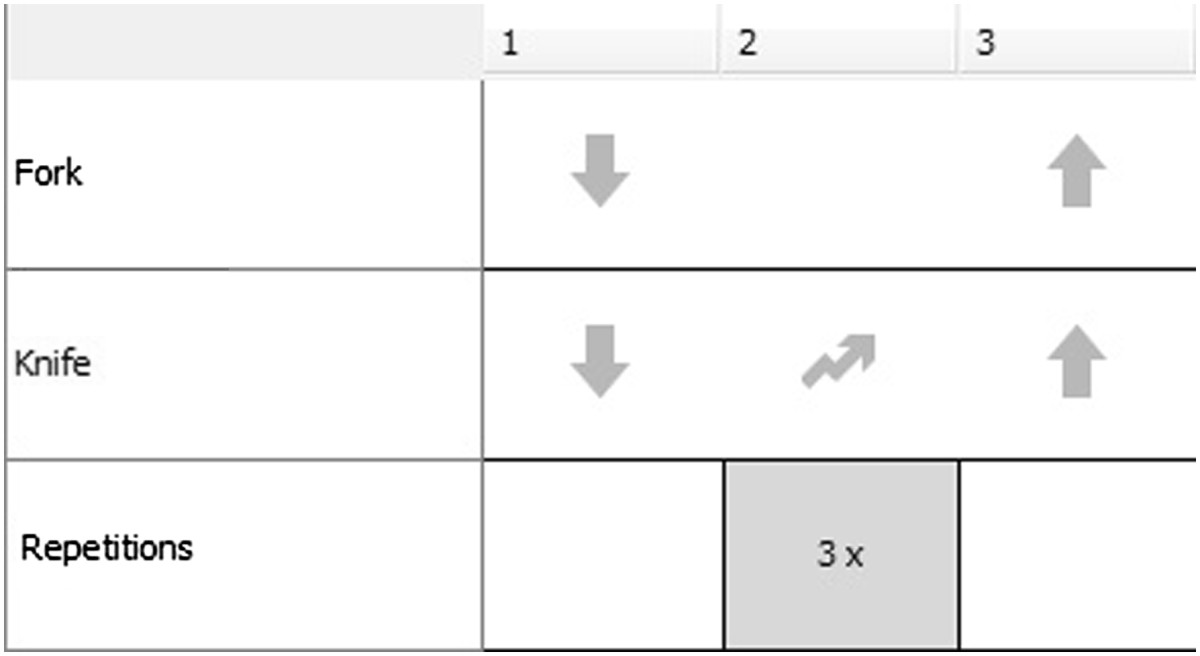
**Example exercise (cutting movement) in the Tag Exercise Creator.**

The current version of the TEC allows therapists to modify and create exercises for the TagTrainer system, by sequencing graphical blocks that represent actions on the TagTile board. Examples of these actions are object manipulations such as placement, movement, and lifting, but also sound playback, breaks, and instructions to the patient. The TEC allows therapists to use any object of their choice within an exercise, and also allows for exercises that make use of multiple objects. Actions are linked to individual objects and therefore allow for bi-manual tasks, such as lifting one object with the left hand, while placing another object with the right hand. An action or a series of actions may be repeated and can be assigned to different objects. For example, an exercise (see Figure 3) might consist of the following steps:

1. The patient places the tips of a knife and a fork on the board.
2. The patient moves the tip of the knife over the board (i.e. cutting movement) three times.
3. The patient lifts the fork and the knife from the board.

Although the TEC has been endowed with an intuitive interface to minimize the chance that therapists make mistakes while creating exercises, it also closely monitors the creation process and immediately notifies therapists of presumed mistakes. For example, if an object placement action is used without specifying the location where the object should be placed, the TEC will notify the user of this missing information. Finally, exercises can be tested directly from within the TEC, both virtually (on screen) and on the TagTile board.

All exercises that are created with the TEC are stored in a central library of exercises and can be found by their name, description, and/or the objects used in the exercise.

### TagTrainer patient interface

While the TagTile Board can be operated as a stand-alone, an auxiliary software application has been developed that runs on an accompanying laptop for communicating information about the exercise progress to the patient. This software, written in Java and called TagTrainer Patient Interface (TTPI), allows therapists to locate existing exercises and compose a tailored training program for each patient consisting of multiple exercises. TagTrainer stores the training history of patients, such that therapists can have an overview of previously executed exercises when composing a new exercise program for a particular patient. Once the exercise program is started, the TTPI gives the patient instructions on what objects are to be used within an exercise and how the exercise should be performed. Finally, the TTPI provides feedback about the patient’s progress on completing the exercises.

### Study design

An action research approach was adopted in the current study with the aim to understand whether and how therapists can act as creators of therapy content using TagTrainer. Action research combines both action and research within the same process and aims at generating knowledge by improving practice and at improving practice by the application of knowledge [21].

A three-week long study was carried out at Adelante Rehabilitation Centre in Hoensbroek, the Netherlands. Three occupational therapists and one physiotherapist (2 m, 2f), all highly specialised in arm-hand training of stroke patients (n = 2) and spinal cord injured patients (n = 2) participated in the study. All participants were considered to be innovators, or early adopters [25] within the environment of the clinic. The therapists participated voluntarily and did not receive any incentive, besides the possibility to learn about and work with a new technology for rehabilitation. The management of the clinic allowed four therapists to be involved in this research, freeing them from part of their regular clinical work to participate. The management was not further involved in the current study and had no influence on the decision whether or not to use TagTrainer. Rehabilitation therapy at the clinic was provided either individually (a therapist treating a single patient) or in a group (a therapist treating multiple patients simultaneously). During group therapy sessions, multiple patients took turns working with TagTrainer.

TagTrainer training was used as part of the regular training program of the patients involved in this study. Note that the decision of whether or not to use TagTrainer for a particular session and patient was left entirely to the therapists, i.e. no patients were pre-selected for the TagTrainer training. Even if the TagTrainer training was suitable for a particular patient, a therapist could still decide to apply a different training method.

The current study has been put before the local Medical Ethics Committee of Adelante in Hoensbroek, the Netherlands. However, as all activities described in this article were part of the patients’ regular rehabilitation programme and no patient data were collected, no a priori ethical approval was necessary. Written informed consent was obtained from the participating therapists. No written consent was obtained from patients, as they were not subjects of the current study and no patient data were collected.

### Study procedure

The study was divided in two consecutive phases dubbed as the ‘use phase’ and the ‘creation phase’ , reflecting the way therapists would use TagTrainer. The use phase lasted for the first week of the study, during which therapists were asked to integrate the TagTrainer system in their daily arm-hand therapy programs. The therapists were free to arrange the TagTrainer system placement according to their own insights, i.e. in any way that they thought would support the arm-hand training. Although they were allowed to modify existing content or create new content for the TagTrainer system, this was not yet actively encouraged. The second phase of the study, the creation phase, lasted for the remaining two weeks. During this phase therapists were actively encouraged, during an introduction meeting organized by the researchers, to become creators of therapy content for the TagTrainer system.

After the study, the two TagTrainer systems were left at the clinic to allow for their continued usage, which supports the original action aim of the study to improve practice, rather than to contend with evaluating the system. Informal contact was maintained with therapists, which served to get an impression of the usage of the system after the end of the study.

### Use phase

During the use phase, which lasted for the first week of the study, several instruction and feedback sessions were planned with participating therapists. First, a one-hour introduction session was conducted with all participants and researchers. During this plenary session, participants were briefed about the goals of the study and their role within the study. After this introduction, feedback sessions with individual therapists were held twice a week for the remainder of the use phase. These meetings lasted for 30 minutes and were used to discuss the progress of the study with the participants. Therapists were encouraged to discuss any problems, requests, or remarks they had concerning the TagTrainer system or the study itself. Additionally, therapists could use these sessions to learn more about using the TagTrainer system. Such technical support to the therapists was also provided outside the scheduled feedback sessions. Researchers were also at hand during therapy sessions in which the TagTrainer system was used for patient treatment. During these sessions, the researchers were able to observe the therapists and the way they worked with the TagTrainer.

### Creation phase

At the start of the second week, another 1-hour introduction meeting was planned. This meeting mainly served to introduce therapists to the modification and creation of therapy content for the TagTrainer system.

Further, two individual meetings per week were scheduled for all therapists. Finally, at the last day of the study, a 1-hour reflection session was scheduled in which all participants (researchers, therapists, and management) were invited to discuss their views on the past three weeks.

### Measures

In their stepwise guidance to facilitate successful implementation of technology in therapy, Hochstenbach-Waelen and Seelen [24] argue that after the initial phases, during which awareness and insight about the new technology are spread amongst the community of therapists, a phase of acceptance follows. During this phase, the attitude, motivation and willingness to change are crucial factors in the success of the implementation process. Since we were interested in the extent to which the implementation process of TagTrainer would develop, the focus of the research efforts has been on measuring these factors and collecting in-depth information about the practices, events, and opinions that underlie these factors.

### Logging

An automated log containing details about the usage of the TagTrainer system was kept. A log entry was added each time an exercise was executed, containing the time of use, the therapist using the system, the pathology of the patient training with the system, and the name of the exercise used. Log entries were also added upon the modification and creation of exercises; these entries contained the name of the exercise and the therapist involved, as well as the date of modification or creation.

### Self-efficacy

Self-efficacy directly affects intent [26]: if a therapist feels that (s)he has not mastered working with the TagTrainer system, (s)he will be less likely to use the system, let alone create new therapy content with it. A self-efficacy questionnaire (based on [19], scaled 0–100, see Table 1) was administered at three moments during the study: right at the start of the study, after one week and at the reflection session at the end of the study. The questionnaire was administered at these three different moments to gauge how long it takes for therapists to feel confident they can use the system correctly. Note that the questionnaire measures therapists’ perception and not actual performance – hence, the questionnaire was first applied at the start of the study, even though therapists had not yet worked with the system.

**Table 1:** **TagTrainer self-efficacy questionnaire**

I’m confident that I can…	Confidence (0–100)
1. Set-up an exercise program with the TagTrainer	
2. Find the right exercises for a given patient	
3. Modify an existing exercise to fit a particular patient	
4. Create an entirely new exercise from scratch	
5. Use the TagTrainer system in individual therapy	
6. Use the TagTrainer system in group therapy	
7. Convince a patient of the usefulness of the TagTrainer	
8. Solve a technical problem with the TagTrainer myself	
9. Help a patient in using the TagTrainer	
10. Use new objects/items for TagTrainer therapy	
11. Convince another therapist to start using the TagTrainer	
12. Help a therapist in using the TagTrainer	
13. Help a therapist in creating new exercises for therapy	

### Technology acceptance

To evaluate acceptance and how it is influenced by increased exposure and familiarity with the system, the UTAUT questionnaire [18] has been administered three times during the study (coinciding with the administration of the self-efficacy questionnaire). UTUAT is a model that examines the relation between people’s acceptance of a technology and several potential determinant factors such as: their behavioural intent (perceived likelihood) to use it, ease of use, usefulness, and social norms. The questionnaire includes 8 subscales (7-point Likert scale), of which only behavioural intent, performance expectancy, effort expectancy, social influence, and facilitating conditions have been shown to be good predictors of acceptance [18].

### Credibility and expectancy

At the end of the study, therapists were invited to give their opinion about the credibility and the expected treatment effectiveness of the provided rehabilitation solution. These factors were measured with a version of the credibility/expectancy questionnaire (CEQ) [27]. This questionnaire includes six questions that asked therapists to indicate their thoughts and feelings about the treatment on offer, on both a 9-point and an 11-point Likert scale. The credibility and expectancy factors (each scoring between 0 and 27) are calculated by aggregating the normalized scores of three questions each, and have high internal consistency and good re-test reliability. In contrast to the other measures that were mentioned before, these measures were only taken once, at the end of the study, as we aimed to solicit their most informed opinion after they have worked with the TagTrainer system.

### Observations

Therapists were observed by the lead researcher (DT) during the full duration of the study, whenever they were using the TagTrainer system. Both written notes and pictures were taken during the observations. The observations focused on the context of use (type of patient, individual or group therapy, etc.), the mode of use (exercises executed, objects used, etc.), and organizational issues (setup of TagTrainer system for therapy, other activities during TagTrainer use, etc.).

### Interviews

Semi-structured interviews were held with therapists during all individual feedback sessions. These interviews covered several topics: the usability of TagTrainer, its use within therapy (times of use, context of use, experiences during usage, etc.), and the structure of the study itself. The (exemplary) testimonials of therapists as used in this paper, though subjective, may give detailed insight to technology developers on how to take into account clinically important issues associated with the (non-) use of technology. They complement objective measures which, being generic and summative, do not provide sufficient insight into the issues underlying the use and non-use of technology on their own.

### Data analysis

Data from the UTAUT questionnaire was analysed with Wilcoxon signed-rank tests. Data from the self-efficacy questionnaire was analysed using paired sample t-tests. Furthermore, select items from the self-efficacy questionnaire (e.g. using TagTrainer in individual therapy vs. group therapy) were compared by performing independent two-sample t-tests. Although the current study has a small sample size, recent research has shown the applicability of t-tests for such sample sizes [28].

Data from observations and interviews were analysed and clustered using affinity diagrams.

## Results

### Logging

**Figure 4:**
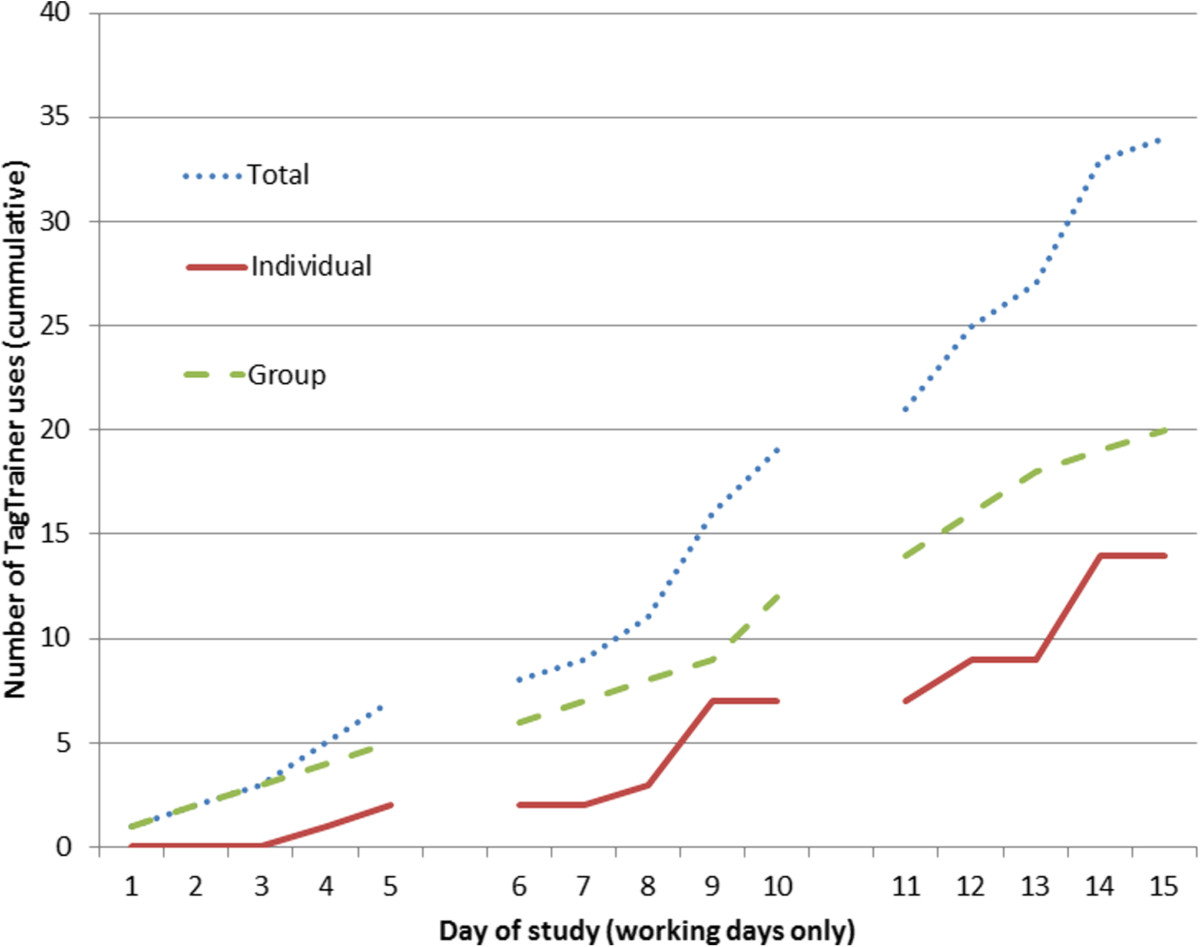
**TagTrainer usage in therapy (working days only, cumulative).** The figure shows that the system has been used more during group therapy than during individual therapy.

The log shows that the TagTrainer system was used in 34 therapy sessions, about 1/4 of all arm-hand training sessions held for the duration of the study. Out of these 34 sessions, 20 sessions were group therapy; the other 14 were individual therapy sessions (see Figure 4). The system was used 25 times in stroke therapy, and nine times in SCI therapy. Thirteen different patients have trained with the system (ten stroke patients and three tetraplegic patients). Note that during some group therapy sessions, multiple patients have subsequently worked with TagTrainer, such that the number of treatments with TagTrainer is slightly higher than the number of sessions in which TagTrainer has been used. In addition, there is not a strict one-on-one relation between patients and therapists; a patient might receive therapy from more than one therapist.

**Figure 5:**
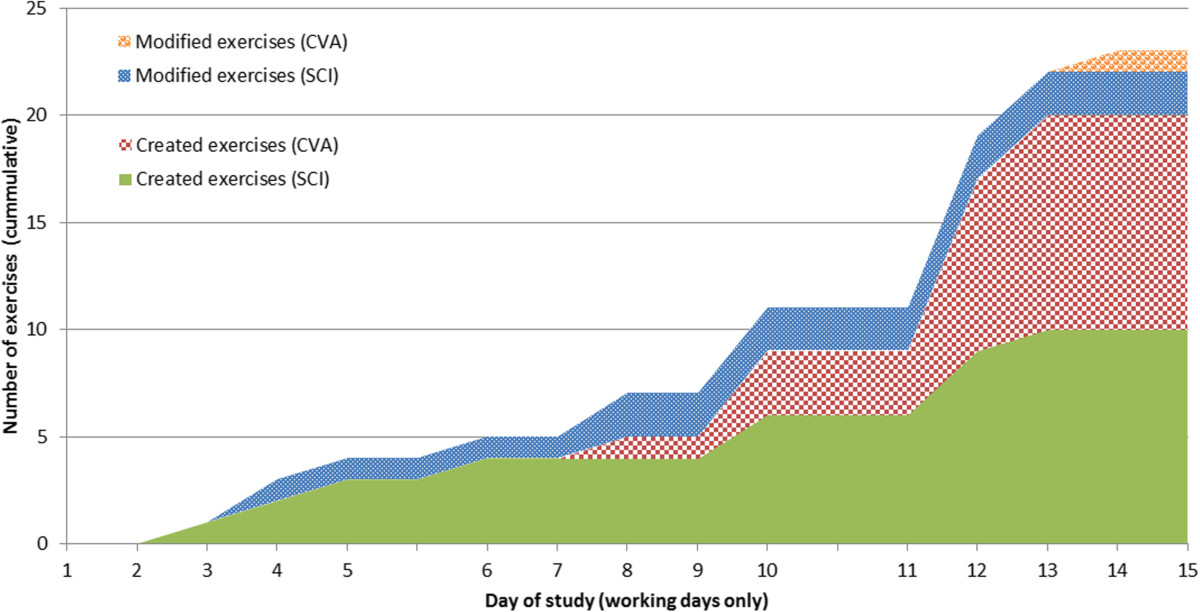
**Number of therapist-created and therapist-modified exercises per pathology (working days only, cumulative).**

All four participating therapists created new exercises for the TagTrainer system. The number of created exercises ranges from two to seven per therapist. In total, 20 new exercises have been created during this study (see Figure 5). In addition to creating new exercises, therapists also modified three existing exercises structurally.

As is visible from Figure 5, exercise creation had started spontaneously before the planned ‘create’ phase of the study had started: one therapist who was curious about the possibilities of the TagTrainer system, in terms of task oriented therapy, decided to create exercises for tasks that were not covered by the default set of training exercises.

### Technology acceptance and self-efficacy

**Figure 6:**
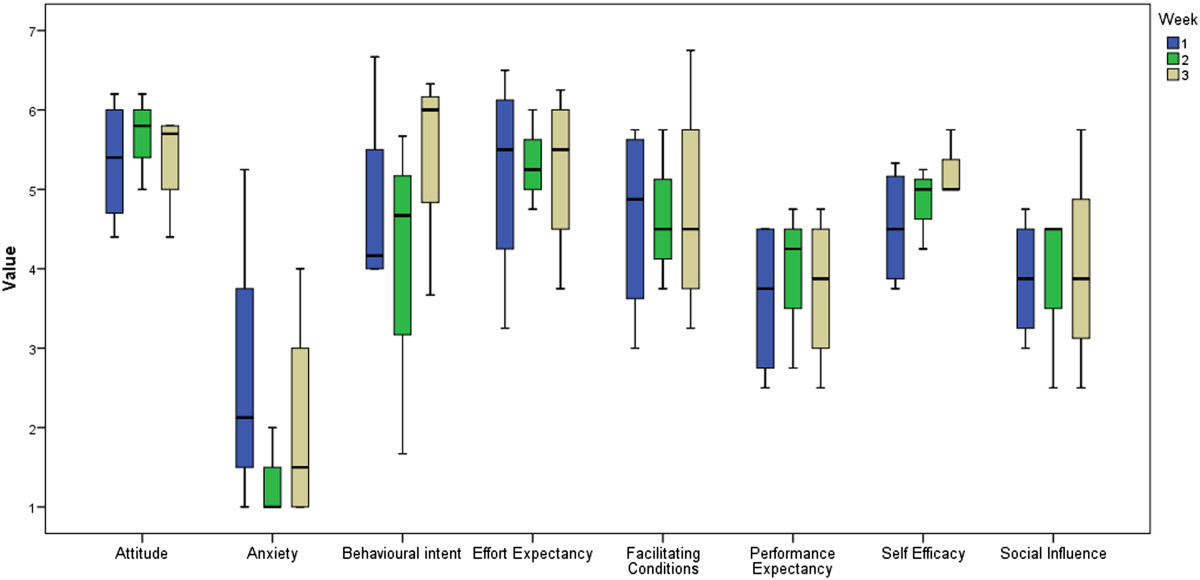
**Boxplots for the UTAUT questionnaire subscales.** Behavioural intent and self-efficacy have increased (not significantly) during the study, but other measures (such as effort expectancy, facilitating conditions and social influence) remained relatively stable over time.

As visible in Figure 6, scores on most subscales of the UTAUT questionnaire are close to neutral apart from anxiety, which is generally low. No significant differences were found between the results from the start and the end of the study. Results from the self-efficacy questionnaire show a significant increase in self-efficacy between the start (M = 52.3, SD = 37.6) and the end (M = 75.4, SD = 19.1) of the study (t (3) = -2.356, p = 0.05). In general, therapists reported moderate to high self-efficacy, except for their perceived ability to resolve technical problems with TagTrainer (M = 32.5, SD = 28.7). In addition, they reported significantly higher levels of self-efficacy (t (3) = 4.899, p = 0.016) for using TagTrainer in individual therapy sessions (M = 80.0, SD = 21.6), compared to group therapy sessions (M = 60.0, SD = 28.3).

### Credibility and expectancy

The credibility (M = 19.5, SD = 3.11) and expectancy (M = 13.9, SD = 5.22) ratings that the therapists gave for the TagTrainer system show that they find it to be credible for arm-hand rehabilitation, but are neutral in respect to the expected effectiveness of the system for the improvement of arm-hand performance. The expected therapeutic value of TagTrainer seems to be corroborated by the scores for performance expectancy of the UTAUT questionnaire. During interviews, therapists mentioned that they were somewhat hesitant to use the TagTrainer system, because it had not yet been clinically validated.

### Observations and interviews

#### TagTrainer usage

Observations and interviews revealed a difference in the way TagTrainer was integrated in the workflow for stroke therapy and SCI therapy. Stroke and SCI treatment take place in two separate rooms that differ in terms of layout, content, and use. The room where stroke therapy is given is relatively crowded with equipment and has many visitors (patients, therapists, and other staff). Therefore, the TagTrainer system was in the room only when it was to be used. When not in use, the system was stored in the clinic’s lab facility. On the contrary, the system that was available for SCI-therapy was placed in the SCI room permanently, regardless of whether it was in use or not. Therapists treating stroke patients indicated that not having TagTrainer available in the treatment room at all times limited their motivation to use it.

### Target population

Therapists agreed in their opinion that the usefulness of the TagTrainer system varies between and within pathologies. For example, stroke patients might be cognitively affected to an extent, which makes it hard for them to work independently with the TagTrainer system. Besides a patient’s cognitive abilities, therapists identified some other factors that influence the suitability of the system for a particular patient. For example, one therapist remarked:

*“I think that patients with low to moderate [arm-hand] function can profit from working with the [TagTrainer] board. For somebody with really good function, the system provides no challenge. The board is simply too small for these high-potentials”.*

Still, the relatively small size of the TagTile board was often mentioned as a limiting factor in the types of exercises that could be executed with the TagTrainer system. In addition, the fact that the current TagTrainer system only supports exercises that are performed on a single board was mentioned as a factor that limited its usefulness:

*“Multiple [TagTrainer] boards would make the system much more useful, it would greatly enhance [our] liberty in creating exercises”.*

### Individual and group use

Although TagTrainer was used more often during group training, one therapist remarked that the system was more suitable for use during individual therapy because patients using the board required 1-on-1 guidance that is not feasible during group therapy. On the contrary, another therapist thought that using the TagTrainer system in group therapy carried an advantage over traditional group therapy methods:

*“By using it [TagTrainer] you force the patient to be more accurate and precise than by just telling somebody ‘put the thing here’. When working with a 5-patient group, you can’t check everyone on their exercise execution. In that light, the TagTrainer system does a better job”.*

Nevertheless, therapists were hesitant to let patients work with the TagTrainer unsupervised. An important requirement for this is that clear feedback should be provided to patients to enable them to carry out exercises and to observe how well they do them. One therapist suggested that:

*“It would be beneficial to patients if long-term feedback (such as accuracy, speed and quality of movements) be collected, so they can compare their performance to previous attempts”.*

Another therapist supported this statement, suggesting that:

*“…four weeks of doing the same exercises won’t work. But if you can show people their progress (in a graphic), it gets interesting”.*

Aside from therapists, patients also seemed to agree on this point. One particular patient was observed motivating himself by trying to perform an exercise as quickly as possible, even though the system did not provide any relevant scores or performance feedback to support this use.

### Content created

Creation efforts ranged from adjustments to existing exercises to the conception and implementation of an entirely new range of exercises. Examples of small adjustments are increasing the size of the target areas in an exercise, modifying the number of repetitions of an exercise, and the use of different objects that are easier for patients to handle.

**Figure 7:**
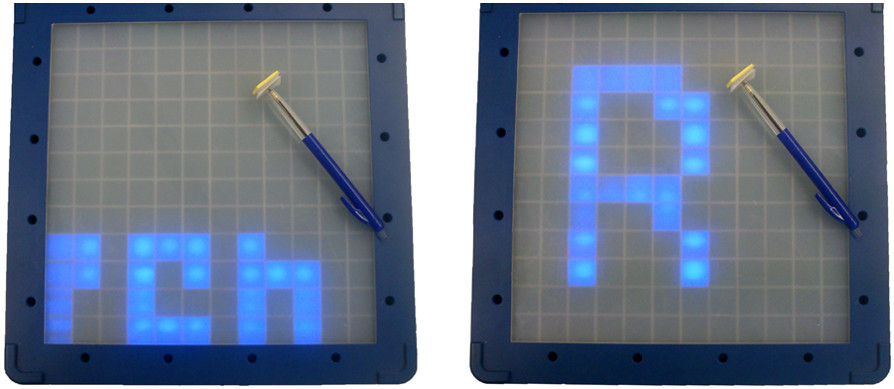
**Two exercises that share a common structure.** The left image shows the original exercise, the right image shows the modified exercise. The modified exercise uses the same object, but lets the patient follow a different pattern on the board.

Therapists also performed modifications that changed the actual structure of an exercise. For example, an exercise that previously demanded a patient to perform pronation and supination in the forearm by rotating a cup was modified to also train ulnar deviation in the wrist. Finally, therapists also created new exercises, entirely from scratch. Some of these exercises were based on already existing exercises, while others represented entirely new ways of exercising. An example of a derivative exercise that was created by a therapist for a particular patient would be when a patient was trained to write the name of his granddaughter (see Figure 7), which was adapted from an exercise featuring a different text.

**Figure 8:**
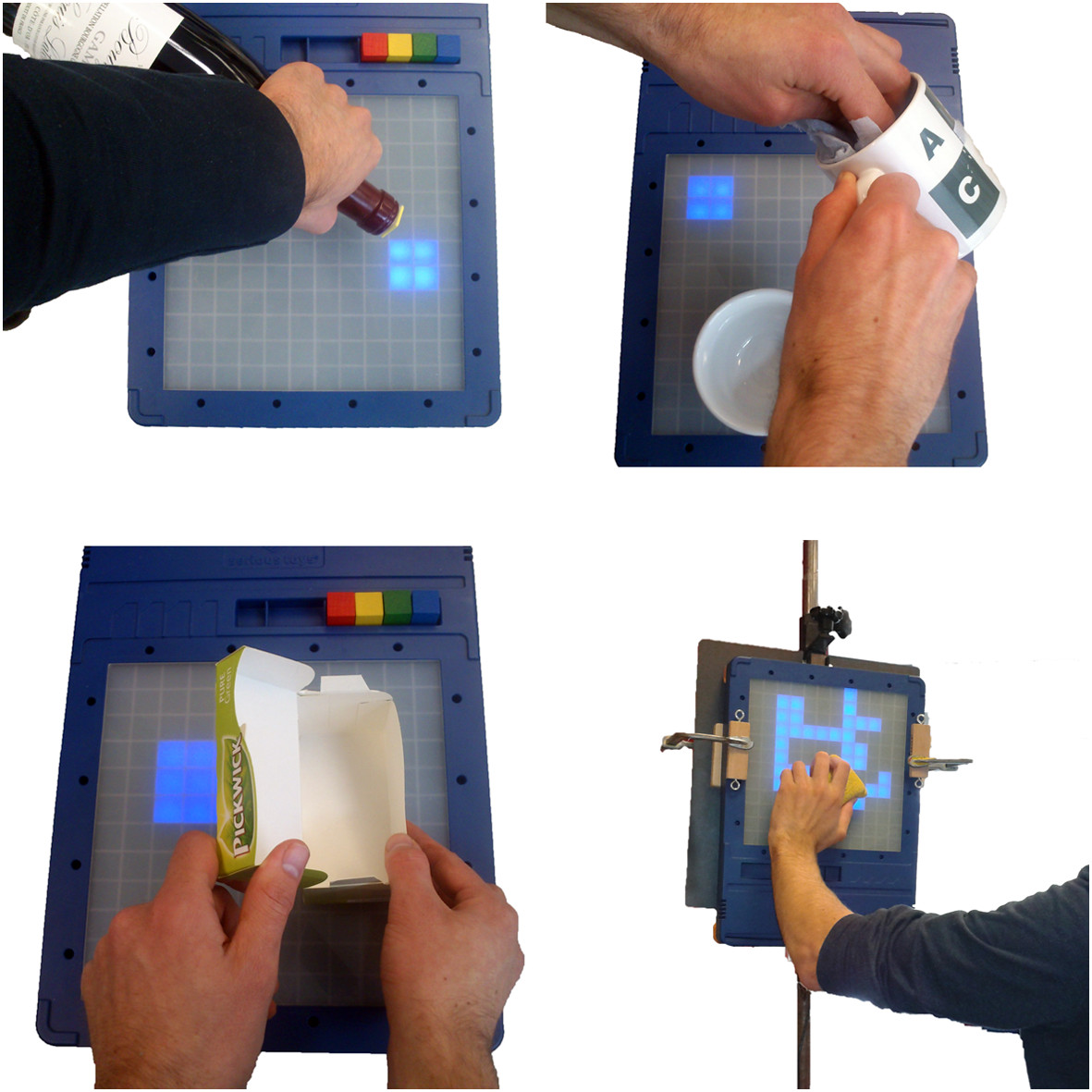
**Four exercises that were created by therapists during the study.** In clockwise order, starting top-left: pouring from a bottle, dishwashing, window cleaning, opening a box of tea.

An example of an exercise that was custom created for a patient from scratch would be when a patient was trained to open a box of tea (see Figure 8, bottom left). In this exercise, tags were placed on the bottom and on the sides of the tea box, as well as on the lid. By equipping the tea box with two tags, the therapist was able to create a complex exercise, challenging the patient to put the tea box down on the board, after which it had to be opened, closed, and finally lifted from the board.

**Figure 9:**
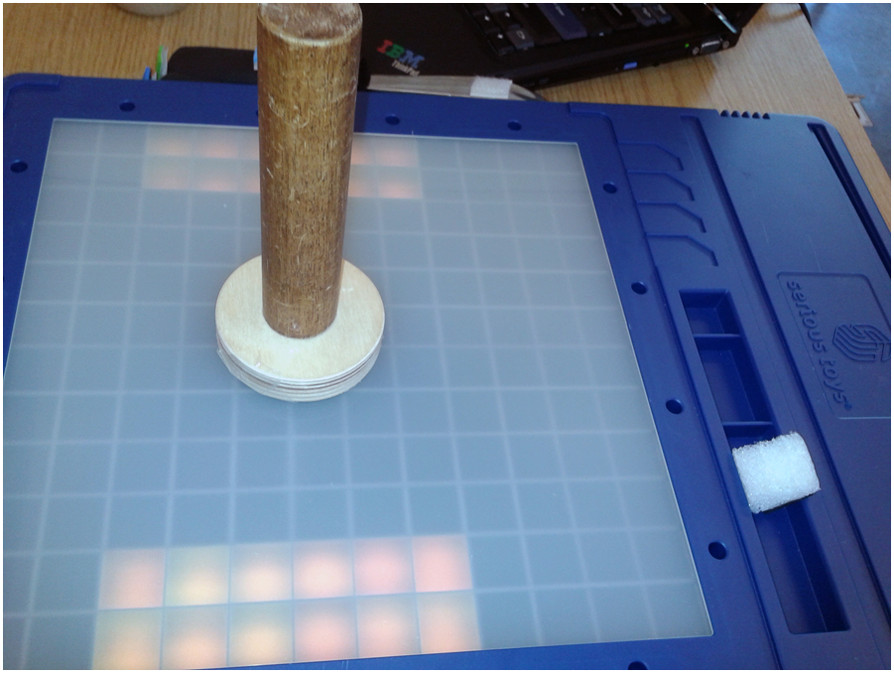
**A custom-made object for the TagTrainer system.** It consists of a cylinder which is mounted on a disc-shaped piece of wood.

Equally relevant is the fact that therapists not only modified and created exercises for the TagTrainer system but created and adjusted physical objects that could be used in therapy. For example, one patient performed a training exercise in which a Velcro strip that was attached around the patient’s wrist had to be moved in a circular shape on the board. However, due to spasticity, the patient constantly performed forearm pronation, which was not intended. In response, the therapist ordered at the clinic’s workshop the production of a cylinder attached to a disc-shaped piece of wood (see Figure 9). This tool had a tremendous effect on the patient’s performance and made certain exercises that were previously inaccessible manageable for the patient.

### Therapist and patient motivation

The act of creation has its influence on the satisfaction that therapists experience from using the TagTrainer system. One therapist explained:

*“I got a feeling of satisfaction when [name of patient] showed his appreciation for the exercise that I had created especially for him”.*

A specific patient was unmotivated to train initially when given the exercises that were included in the initial setup of TagTrainer, describing them as *“childish and not fun to use”*. Suspecting this was due to the generic nature of the exercises, the therapist developed for the following training session two exercises custom tailored to this particular patient. As the therapist recalls:

*“The patient initially was not motivated, so I asked him: what do you want? He wanted to train in preparing sandwiches. At that time, he wasn’t doing this at all, so I took a piece of foam and a knife, and created the exercise. He told me after the weekend, that for the first time in 9 months he had prepared his own sandwiches! I’m not sure whether this has caused it, but suddenly his motivation had increased”.*

Another source of motivation for therapists was the creations of their colleagues. Upon witnessing the newest creation of his colleague, a therapist jokingly remarked:

*“That looks impressive, of course now I have to beat [your creation]…”.*

Although the remark was made as a joke, later that day the therapist actually sat down to create a new exercise for his patient.

### Sharing of created exercises

Therapists had one TagTrainer system available per pathology, so they always had to share their system with a colleague. Since exercises were stored locally, per system, this meant that *all* created and modified exercise content was there to be shared amongst therapists for a given pathology, without them having to take any explicit action.

Explicit sharing, consciously initiated by therapists towards their colleagues, happened only on a modest scale during the study. Especially in the stroke domain, the therapists were often unaware of the exercises that their colleagues had modified or created. However, in the SCI domain, the participating therapists regularly witnessed occasions in which the other therapist was applying a self-created exercise in therapy. Apart from informing them about the availability and application of new exercises, this stimulated them to create new exercises themselves.

### Exercise reuse

Therapists found it hard to identify the exact nature of the exercises that were available in the system. For example, one of the therapists remarked:

*“I find it difficult to use the [TagTrainer] system in therapy, since I don’t know exactly what all of the exercises entail. Just a name and a description are not enough for me to estimate, for example, how long a patient would take to complete an exercise”.*

The fact that it is hard to capture the exact nature of an exercise in just a couple of words had also influenced the way therapists approached content that was produced by colleagues. One therapist remarked:

*“I hardly try out any new exercises. I’d like to, but I feel that I shouldn’t experiment with this during a patient’s therapy time”.*

This touches on another, more general issue that was named by all therapists during the study: time shortage. In the current situation, the clinic does not allocate time for therapists to work on things that are not directly beneficial to a patient’s rehabilitation process. In other words, although creating a new exercise might *eventually* contribute to a patient’s rehabilitation, it is not *directly* beneficial and hence, strictly speaking, not part of the work that therapists are expected to perform. Still, the therapists managed to create 20 new exercises, mostly during the time that was reserved for feedback on the study, as well as during breaks and after-hours.

## Discussion

TagTrainer is a technology for upper limb training that allows for exercise variability and tailoring. The study focused on the acceptance of TagTrainer by therapists and the extent to which they can adjust their practices to assume the role of exercise author that exercise ‘tailorability’ requires.

### Acceptance and self efficacy

Therapists indicated lower self-efficacy for TagTrainer use in group training compared to individual training. This seems to be related to the amount of support that patients working with TagTrainer currently require – in group sessions, such support is not permanently available. However, in this respect, when anticipating future improvements therapists envisioned TagTrainer as a suitable technology for group therapy sessions. TagTrainer could monitor a patient’s performance, providing therapists with extra time to attend other patients.

During this study, the therapists’ self-efficacy and their behavioural intent increased. The increase in self-efficacy indicates that therapists can easily learn to use TagTrainer. The increase in behavioural intent suggests that after an initial training period, TagTrainer reached satisfactory acceptance among therapists.

On the other hand, the measures on performance expectancy, effort expectancy, and social influence showed virtually no variation over time. This was to be expected regarding social influence, since the social context of use remained constant for the duration of the study. One would however have expected effort expectancy to decrease, since TagTrainer was adapted during the study to better fit the needs of the therapists. The low scores on anxiety are a positive indication for the system though it could be attributed to the specific therapists who are early adopters.

### Creation process

To the best of the authors’ knowledge this study is the first to examine and demonstrate the feasibility of putting therapists in the role of authors of training exercises for rehabilitation technology. Previous research points at the importance of patient customized exercises [8] and sufficient exercise variability [8, 12], which necessitates this role shift, but until now no such technology has been implemented as part of daily rehabilitation practices and the factors that will enable this shift are not to this point understood. Exercise adjustment in related systems (e.g., [29]) typically pertains to the tailoring of parameters of movements and targets rather than defining the very purpose and nature of an exercise as is the case in the current study.

Overall, the results presented show clearly that therapists can act as creators of training content. On the downside, they did not re-use existing content as much as expected. It had been anticipated that therapists would re-use exercises to save effort, and this was expected to be a way to pool resources and achieve an economy of scale. In practice, some of the exercises created during this study were very similar to exercises already available. Therapists perhaps were hampered by the sheer number of available exercises (about 150 at the start of the study), which they did not have the time to explore and get acquainted with. Perhaps regular use of TagTrainer would resolve this issue, eventually allowing therapists to familiarize even with much larger sets of exercises. From a system perspective it appears that auxiliary tools need to be developed to support the browsing of exercises, standardized descriptions of exercises, versioning, and other (visual) means of displaying an exercise’s content and nature.

During this study, a difference emerged in the way in which therapists from different pathologies shared exercises. One possible explanation for this difference is the fact that the therapists working in the SCI domain were often present in the same room when one of them was working with the TagTrainer system. Exercises that one therapist was using, were thus visible to the other therapist. Another factor that may have influenced the amount of sharing is the number of patients that received TagTrainer therapy. The stroke rehabilitation group used the system with 10 different patients, while the SCI group used it with only three different patients. Because of the limited number of different patients that were treated in the SCI group, the number of sessions with the TagTrainer system per patient was higher than for the stroke group. Because the history of performed exercises was kept for each patient, therapists could easily see which exercises the patient had previously been doing with the other therapist. It is likely that this positively influenced the reuse of exercises by therapists other than the original creator.

It is also interesting to note that cross-pathology sharing of exercises was not observed during this study. Since the two TagTrainer systems used in this study were not mutually connected, sharing across pathologies was not explicitly facilitated. However, given the responses of therapists during plenary feedback sessions in which they exchanged their experiences on creating exercises, exploration of the option to facilitate the sharing of exercises in a wider context seems valuable.

Previous research has indicated that insufficient time is a major barrier for therapists regarding the authoring of training content [17]. Although the therapists found enough motivation to create exercises outside of their paid hours, this solution is neither sustainable nor scalable. From an organizational perspective, management can support this shift by allocating time on creating and tailoring exercises. From a system design perspective, rather than looking solely at easy to use authoring tools and improving usability, a broader range of solutions need to be considered in future research. One solution that has been successful in the domains of open source software development, Wikipedia, and other examples of crowd sourcing, is to establish and support a so-called culture of participation [30]. In such a culture of participation, a community that is centred on the TagTrainer platform would collaboratively maintain, extend, and modify available content to make it of optimal use for rehabilitation therapy. This community would consist primarily of therapists, but should also include stakeholders such as (clinical) technicians and even patients, extending across multiple clinics, thus allowing rehabilitation centres to pool solutions and resources.

### Application to different pathologies

Thus far, TagTrainer has only been used as a part of the upper extremity rehabilitation of stroke and spinal cord injured patients. Both patient groups suffer from paresis, muscle spasticity, and coordination problems. However, this way of technology-supported task-oriented arm-hand training might also be very useful in the rehabilitation of other neurological diseases with similar arm-hand performance impairments, especially when task-oriented training has been found beneficial, such as in multiple sclerosis [31] and in cerebral palsy [32]. Investigations into the feasibility of using the TagTrainer for arm-hand rehabilitation in these pathologies are ongoing.

### Study limitations

Although the present study resulted in valuable information, which was collected over the relatively short time period of three weeks, it is expected that more insights into the adoption of TagTrainer could have been gained in a longer study and in more sites. The authors have planned a longer follow-up study in multiple sites to address this limitation.

## Conclusions

Tailorability and variation of rehabilitation exercises can be achieved by providing dedicated tools that enable therapists to create and modify them easily. This paper introduced TagTrainer, a system that supports the creation and modification of arm-hand training exercises that are executed on an interactive board, with every day physical objects augmented with RFID tags. TagTrainer was used as part of therapy offered to patients after stroke and spinal cord injuries during a three-week period, during which therapists were able to learn and use the system effectively. With TagTrainer, therapists assumed radically new professional roles as authors, editors, and potential publishers of training content. This study has shown that this role switch is feasible, though it was found to be contingent on several contextual factors, such as organizational support, physical and social environment, support by peers, as well as technological factors, such as ease of use and availability of tools to monitor patient performance and progress. Future work will focus on how content authoring and sharing practices can be supported and sustained for longer periods.

## Authors’ original submitted files for images

Below are the links to the authors’ original submitted files for images. Authors’ original file for figure 1 Authors’ original file for figure 2 Authors’ original file for figure 3 Authors’ original file for figure 4 Authors’ original file for figure 5 Authors’ original file for figure 6 Authors’ original file for figure 7 Authors’ original file for figure 8 Authors’ original file for figure 9 Authors’ original file for figure 10 Authors’ original file for figure 11 Authors’ original file for figure 12 Authors’ original file for figure 13 Authors’ original file for figure 14
