# Supplementary figures and images for: TagTrainer: supporting exercise variability and tailoring in technology supported upper limb training
Source: J Neuroeng Rehabil. 2014 Sep 24;11:140. doi: 10.1186/1743-0003-11-140 (PMC4182772; doi:10.1186/1743-0003-11-140)

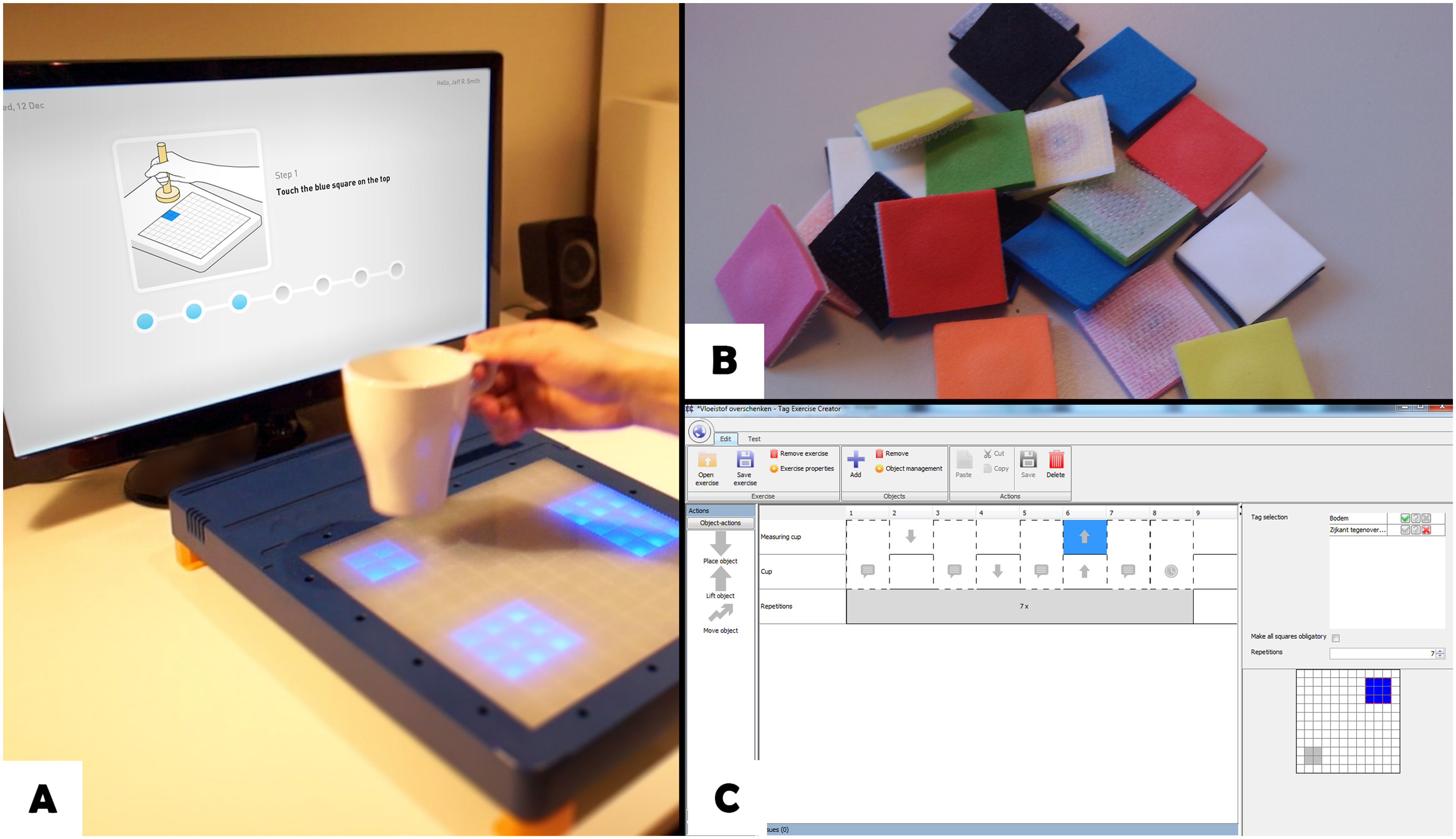

Supplement: Supplementary file 1 — Authors’ original file for figure 1 [file 12984_2013_660_MOESM1_ESM.tif]

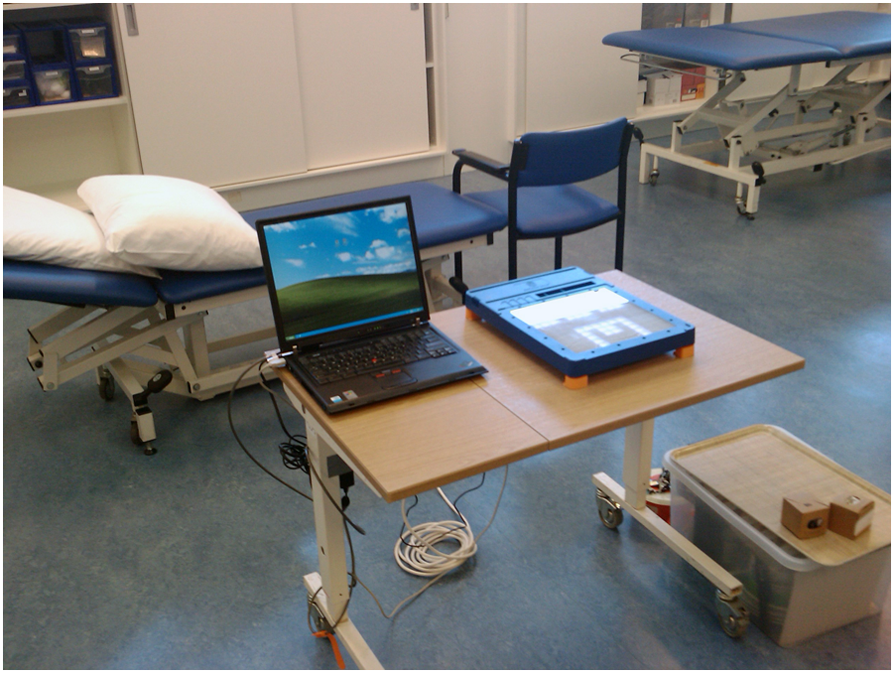

Supplement: Supplementary file 2 — Authors’ original file for figure 2 [file 12984_2013_660_MOESM2_ESM.tif]

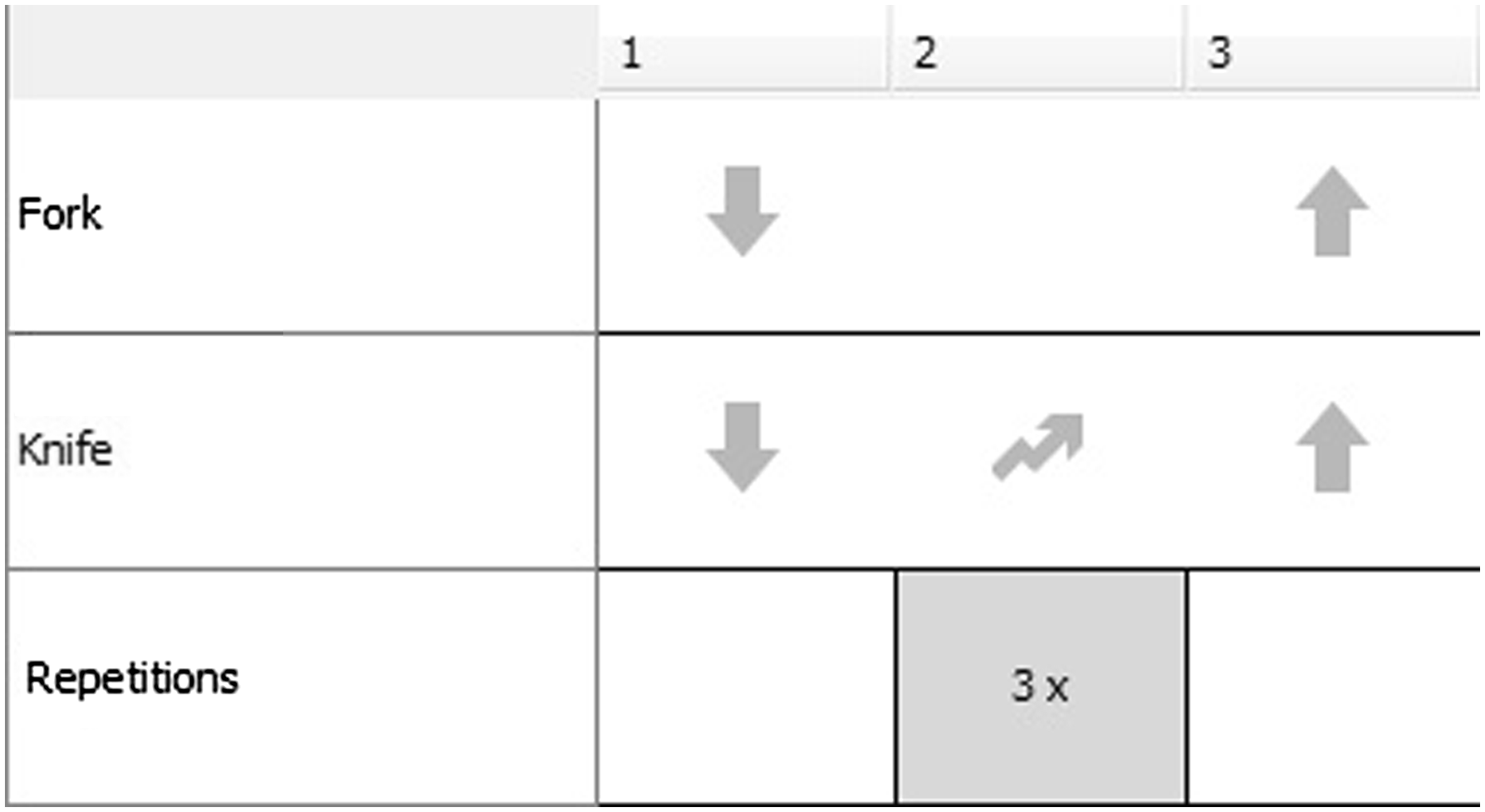

Supplement: Supplementary file 3 — Authors’ original file for figure 3 [file 12984_2013_660_MOESM3_ESM.tif]

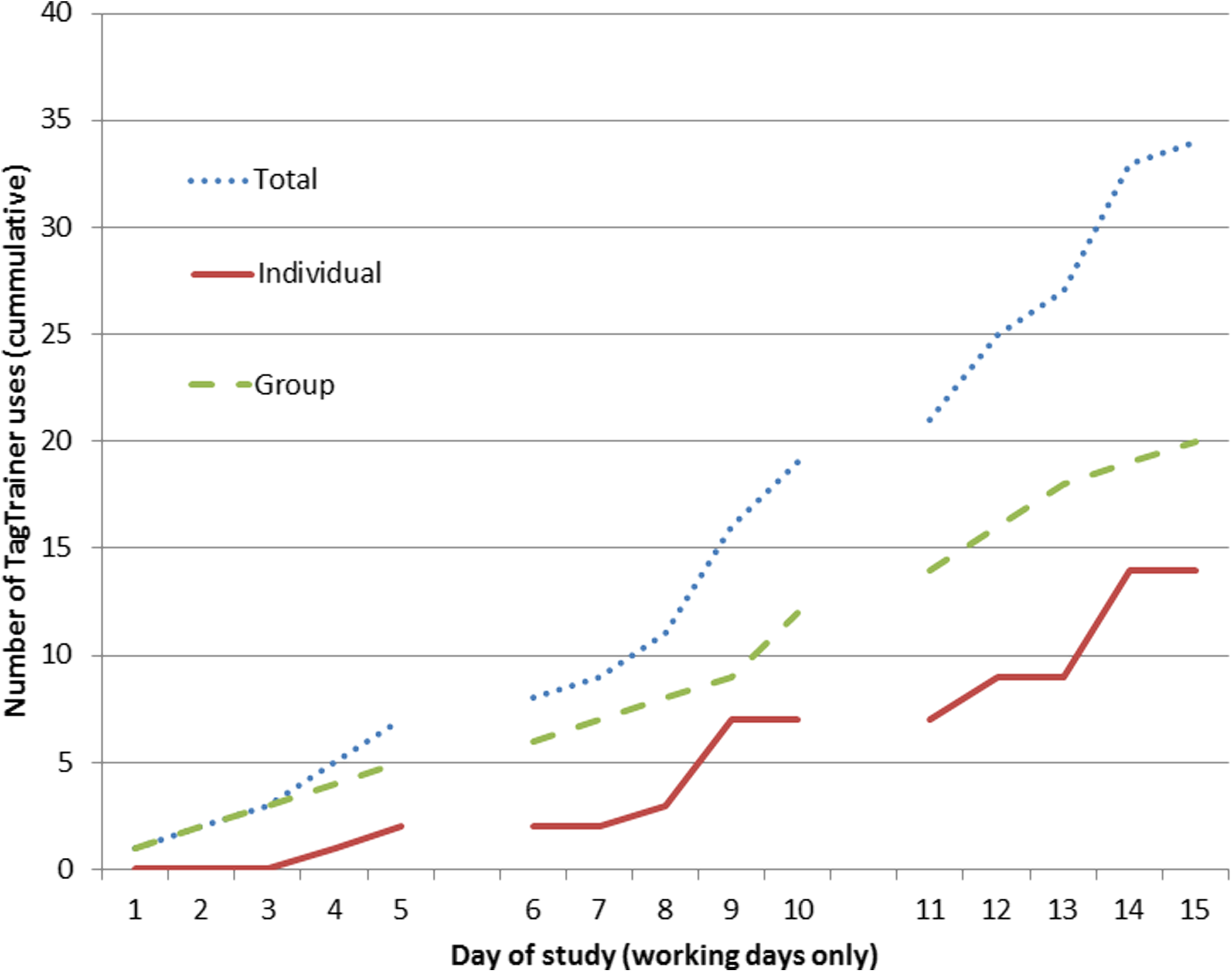

Supplement: Supplementary file 4 — Authors’ original file for figure 4 [file 12984_2013_660_MOESM4_ESM.tiff]

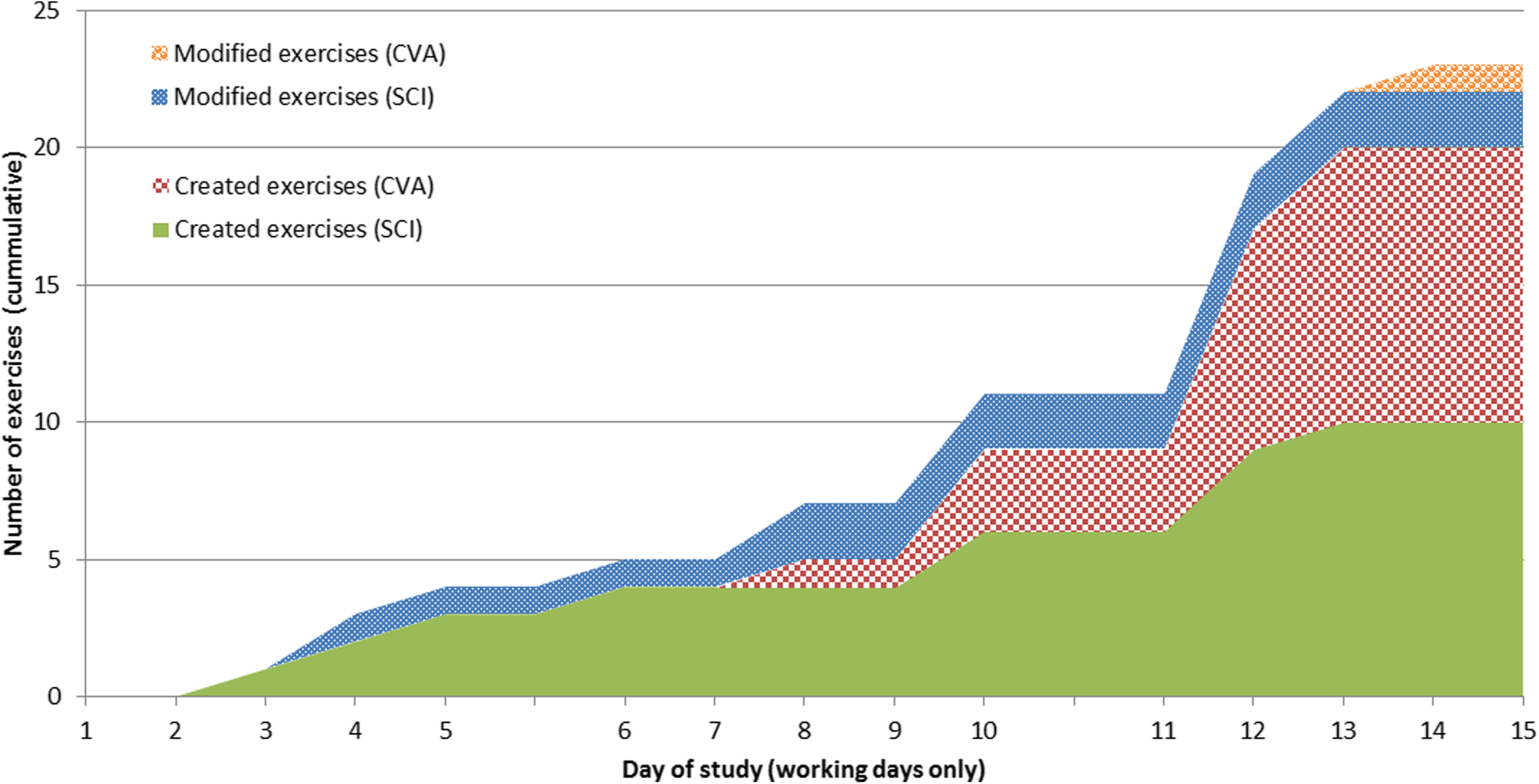

Supplement: Supplementary file 5 — Authors’ original file for figure 5 [file 12984_2013_660_MOESM5_ESM.tiff]

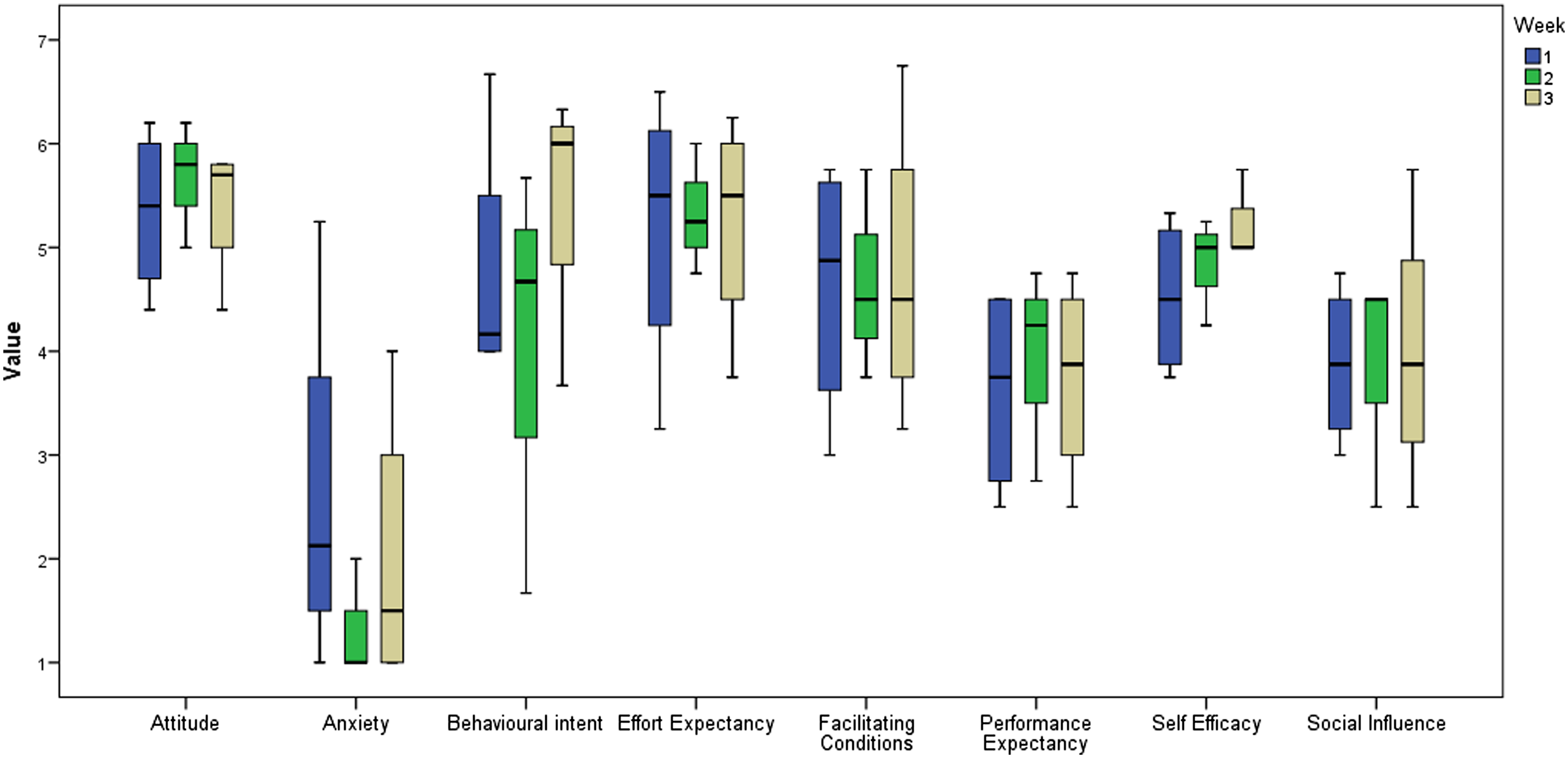

Supplement: Supplementary file 6 — Authors’ original file for figure 6 [file 12984_2013_660_MOESM6_ESM.tif]

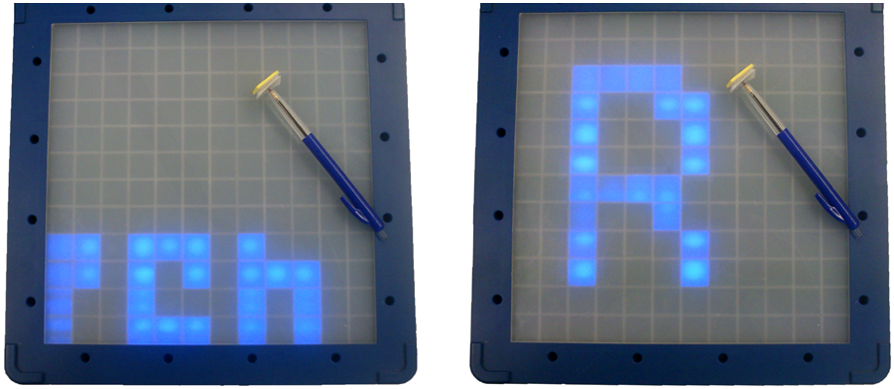

Supplement: Supplementary file 7 — Authors’ original file for figure 7 [file 12984_2013_660_MOESM7_ESM.tif]

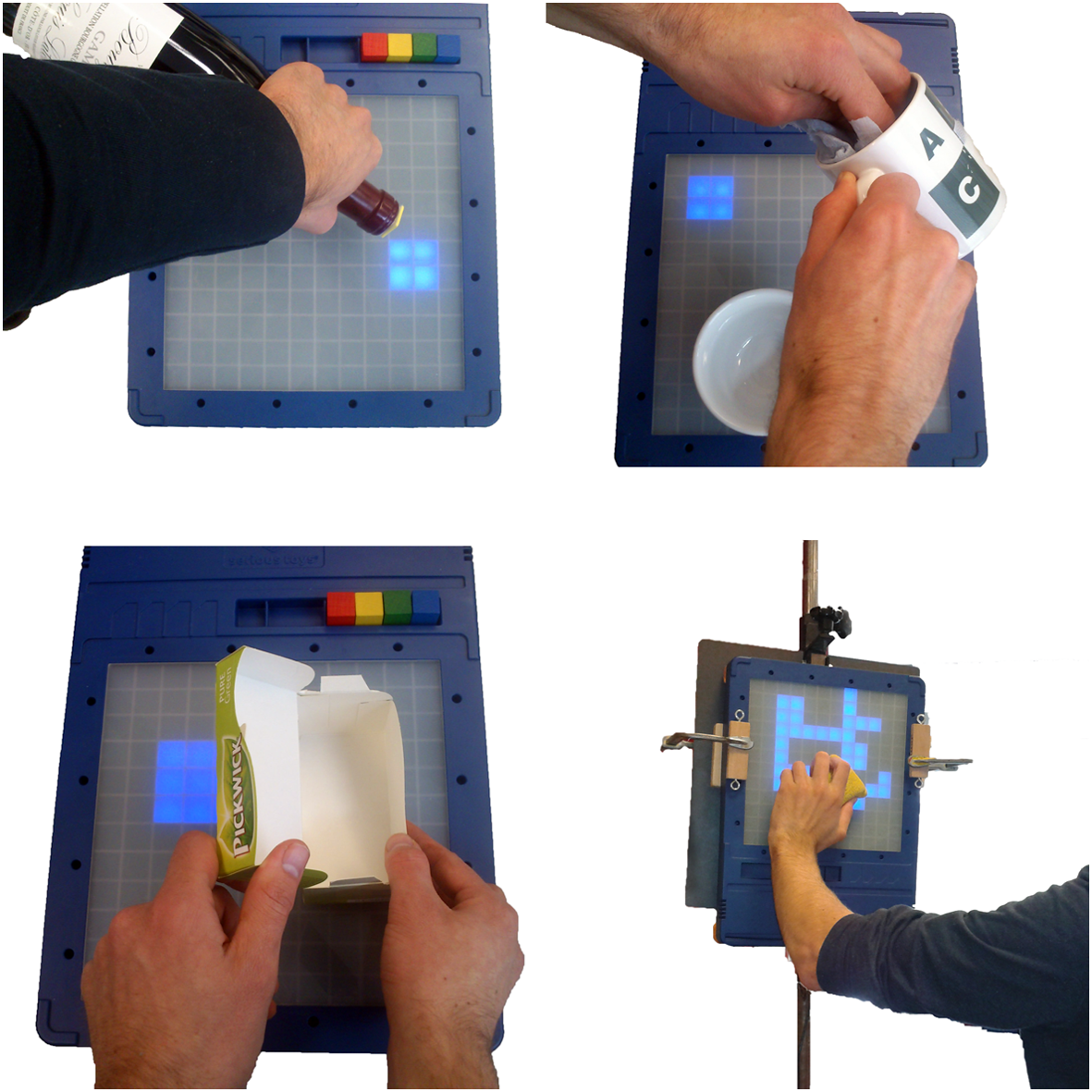

Supplement: Supplementary file 8 — Authors’ original file for figure 8 [file 12984_2013_660_MOESM8_ESM.tif]

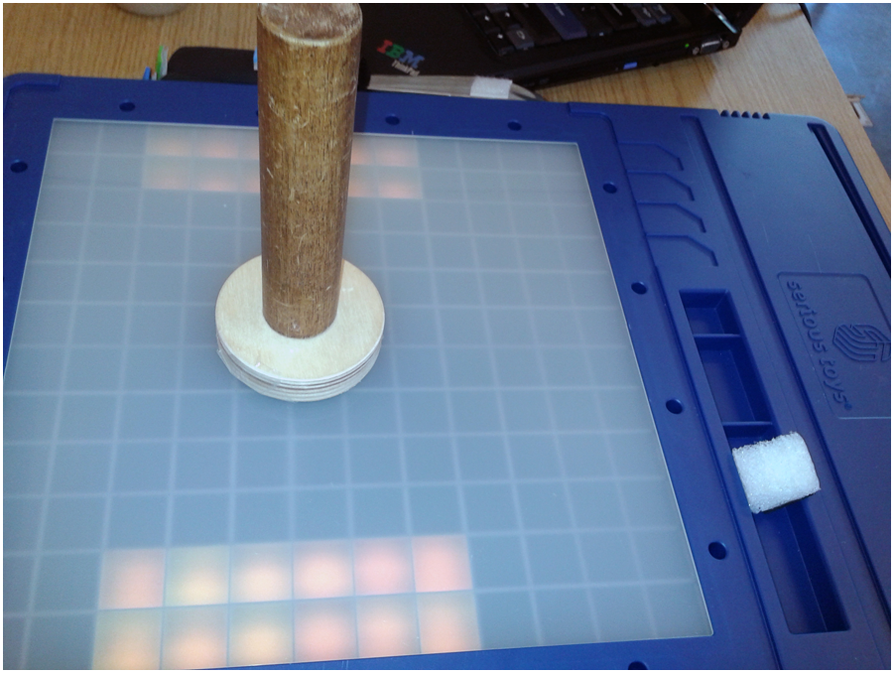

Supplement: Supplementary file 9 — Authors’ original file for figure 9 [file 12984_2013_660_MOESM9_ESM.tif]

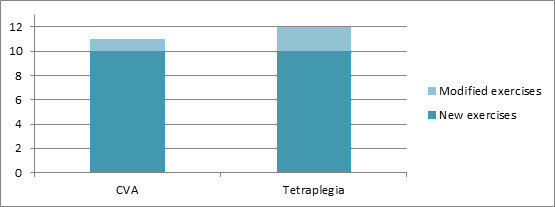

Supplement: Supplementary file 10 — Authors’ original file for figure 10 [file 12984_2013_660_MOESM10_ESM.png]

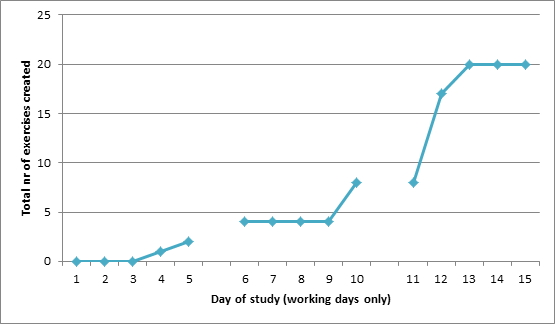

Supplement: Supplementary file 11 — Authors’ original file for figure 11 [file 12984_2013_660_MOESM11_ESM.png]

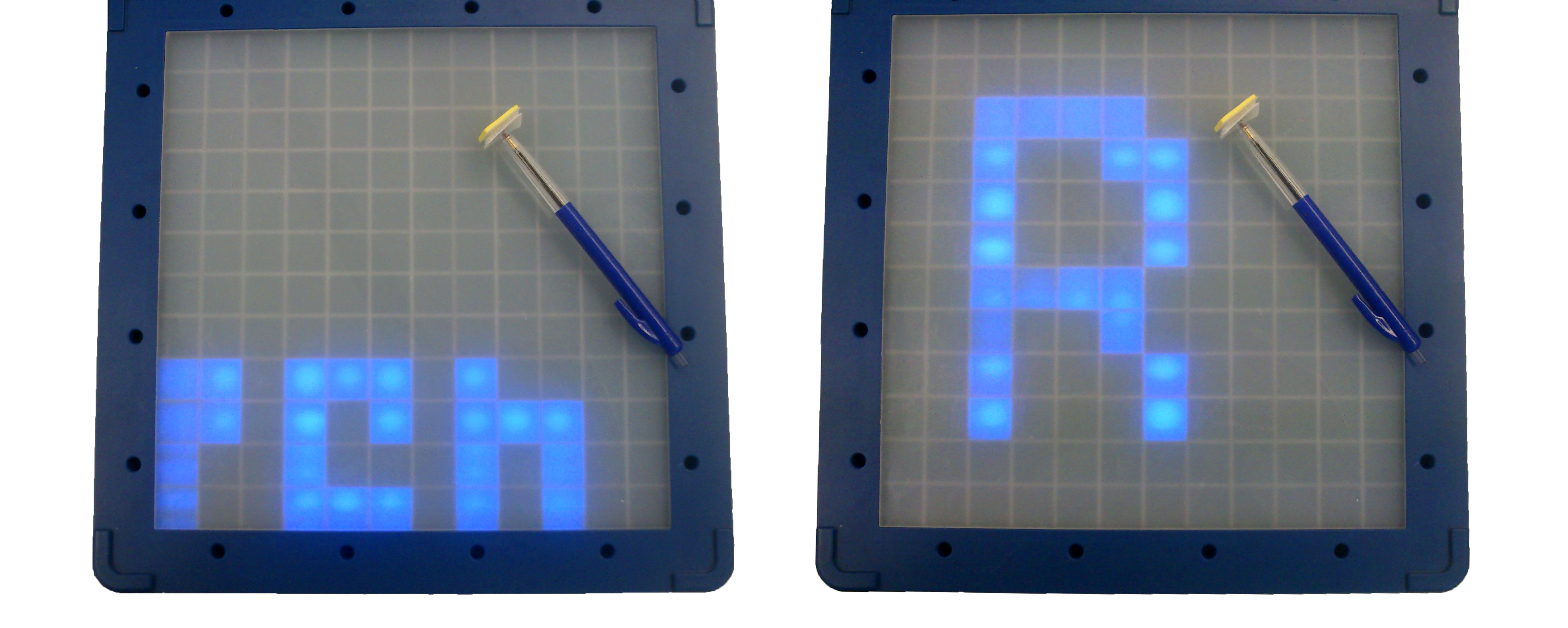

Supplement: Supplementary file 12 — Authors’ original file for figure 12 [file 12984_2013_660_MOESM12_ESM.png]

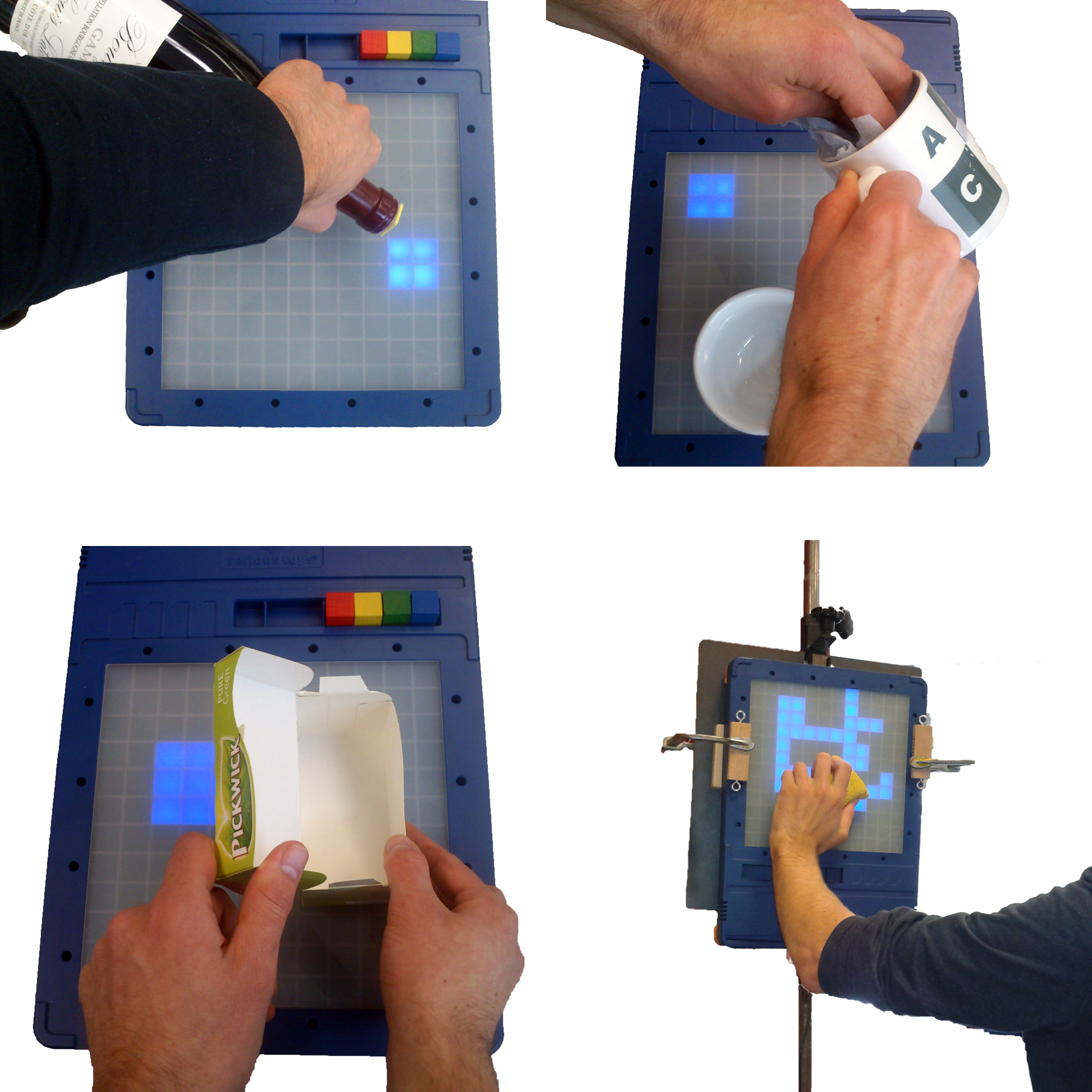

Supplement: Supplementary file 13 — Authors’ original file for figure 13 [file 12984_2013_660_MOESM13_ESM.png]

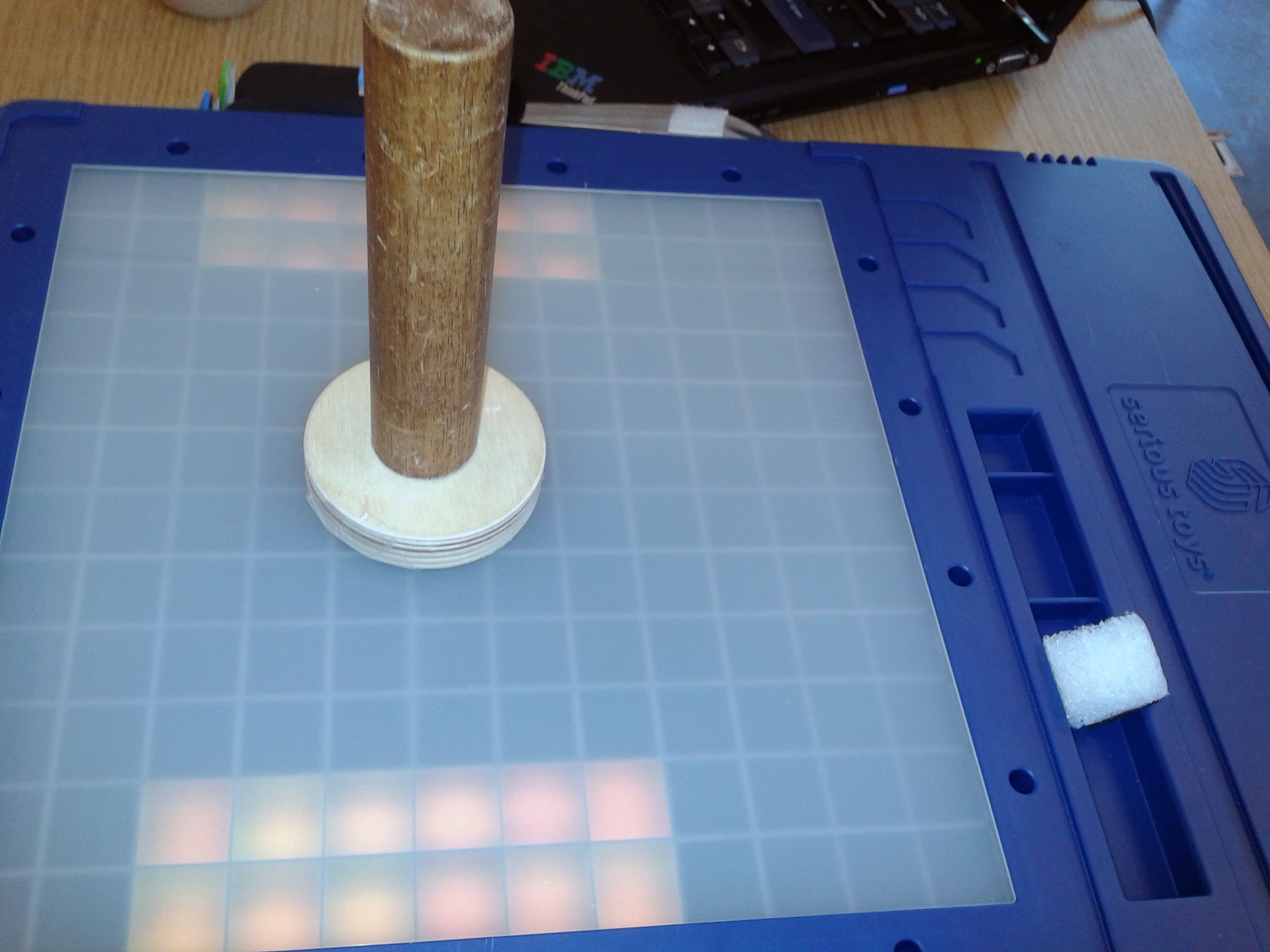

Supplement: Supplementary file 14 — Authors’ original file for figure 14 [file 12984_2013_660_MOESM14_ESM.jpeg]
